# Immune changes induced by periampullary adenocarcinoma are reversed after tumor resection and modulate the postoperative survival

**DOI:** 10.1007/s12672-023-00768-2

**Published:** 2023-08-23

**Authors:** Anna Landerer, Ruth Himmelsbach, Esther A. Biesel, Stefan Fichtner-Feigl, Uwe A. Wittel, Sophia Chikhladze

**Affiliations:** 1 Department of General and Visceral Surgery; Medical Center and Faculty of Medicine, University of Freiburg, Hugstetter Strasse 55, 79106 Freiburg, Germany; 2 Department of Medicine, Cedars-Sinai Cancer Institute, Cedars-Sinai Medical Center, 8700 Beverly Blvd, Los Angeles, CA 900048 USA

**Keywords:** Adenocarcinoma, Pancreatic cancer, Pancreatoduodenectomy, Perioperative course, Immune response, Survival

## Abstract

**Background:**

Tumor growth encompasses multiple immunologic processes leading to impaired immunity. Regarding cancer surgery, the perioperative period is characterized by additional immunosuppression, which may contribute to poorer outcomes. In this exploratory study, we assessed plasma parameters characterizing the perioperative immunity with a particular focus on their prognostic value.

**Patients and methods:**

31 patients undergoing pancreatoduodenectomy were enrolled (adenocarcinoma of the pancreatic head and its periampullary region: n = 24, benign pancreatic diseases n = 7). Abundance and function of circulating immune cells and the plasma protein expression were analyzed in blood samples taken pre- and postoperatively using flow cytometry, ELISA and Proximity Extension Assay.

**Results:**

Prior to surgery, an increased population of Tregs, a lower level of intermediate monocytes, a decreased proportion of activated T-cells, and a reduced response of T-cells to stimulation in vitro were associated with cancer. On the first postoperative day, both groups showed similar dynamics. The preoperative alterations did not persist six weeks postoperatively. Moreover, several preoperative parameters correlated with postoperative survival.

**Conclusion:**

Our data suggests systemic immunologic changes in adenocarcinoma patients, which are reversible six weeks after tumor resection. Additionally, the preoperative immune status affects postoperative survival. In summary, our results implicate prognostic and therapeutic potential, justifying further trials on the perioperative tumor immunity to maximize the benefit of surgical tumor therapy.

**Supplementary Information:**

The online version contains supplementary material available at 10.1007/s12672-023-00768-2.

## Introduction

Solid cancer is one of the leading causes of death worldwide. The field of immuno-oncology is a major area of current research, with immunotherapeutic concepts emerging as a substantial strategy. At first, they were mainly employed in non-resectable advanced stages, but have recently found their way into adjuvant and neoadjuvant regimes [1, 2]. Since Rudolf Virchow described infiltrating immune cells in neoplastic lesions in 1863 [3], the interaction between cancer, inflammation and the host immunity has been the subject of intense research. Today, tumor-promoting inflammation and the avoidance of immune destruction are counted among the hallmarks of cancer [4]. In particular, the tumor microenvironment and its inflammatory and immunologic components are widely described [5]: inflammation-promoting cytokines and chemokines are released at the tumor site and induce leucocytic infiltration and activation. Infiltrating immune cells, mainly cells of the innate immune system like macrophages, that differentiate from circulating monocytes and neutrophils, but also lymphocytes, initiate an inflammatory response producing apoptosis-inducing and cytotoxic mediators e.g. reactive oxygen species (ROS), tumor necrosis factor α (TNFα), various interleukins (e.g. IL1ß, IL6, IL10) and growth factors. Persisting immune activation leads to a chronic inflammatory state, creating an environment which inhibits an effective anti-tumor response and promotes tumor growth and metastasis [4, 6]. Moreover, systemic immunologic changes are confirmed. In pancreatic cancer (PC), a decreased number of circulating CD8^+^ cytotoxic T-cells, an elevated count of regulatory T-cells (Tregs), and higher serum levels of potentially immune-regulatory cytokines like IL6, IL10 or TGFß1/2 have been found in peripheral blood [7–9]. Such findings are associated with advanced disease and reduced survival [7, 10, 11]. Monocytes, representing the innate immunity, have been shown to correlate with poor outcomes in various cancer entities [12, 13], but there is a lack of evidence on monocytes and their functional subsets in PC.

Regarding cancer treatment, the surgical resection of the primary tumor poses one of the main curative approaches in early tumor stages. But while the tumor itself generates a complex interaction with the host immune system, the perioperative period is additionally characterized by immunological processes commonly described in the concept of systemic inflammatory response syndrome (SIRS), followed by a compensatory anti-inflammatory response syndrome (CARS) [14]: tissue injury, caused by surgery, releases damage-associated molecular patterns (DAMPs), inducing a systemic inflammatory response with an upregulation of the innate immune system, an imbalance of pro- and anti-inflammatory mechanisms and an impaired cell-mediated immunity [14, 15]. As a result, surgical resection has been described as an ‘immunological hit,’ compromising the anti-tumor immunity with a susceptible postoperative period that may facilitate tumor expansion [16, 17]. A previous study has shown a higher risk for tumor recurrence in patients with colorectal cancer when they underwent open colectomy compared to that of laparoscopic-assisted colectomies, which are associated with reduced tissue trauma [18]. Moreover, the development of postoperative surgery-related complications like anastomotic insufficiency or pancreatic fistula was associated with a higher risk of tumor recurrence and an increased mortality rate [19–21]. These findings provide clinical evidence for the importance of an effective anti-tumor response in the perioperative period, especially regarding remaining residual and circulating neoplastic cells [17].

A better understanding of the perioperative immunity in cancer patients undergoing surgical tumor resection could provide targets for prognostic markers and therapeutic options. To study the surgical impact on immune systems that may already be impaired by the tumor, we performed an exploratory multiparametric prospective analysis comparing systemic immunologic variables in the perioperative course between patients with adenocarcinoma and benign pancreatic diseases, with both undergoing pancreatoduodenectomy (PD). We evaluated numeric and functional alterations of peripheral leucocyte populations and the plasma expression of potential protein biomarkers with a special reference to their effect on overall survival in patients with malignant disease.

## Materials and methods

### Subjects and sampling

Within the period of one year, we performed a cohort study of 38 consecutive patients with the indication for PD at the Department of General and Visceral Surgery at the University Medical Center Freiburg, who met the inclusion criteria (good general condition, age > 18 years and written informed consent). Patients with chronic inflammatory diseases (e.g. chronic pancreatitis) or with tumor entities other than adenocarcinoma (e.g. neuroendocrine tumors) were excluded from the study cohort. To determine perioperative changes, venous whole blood and plasma samples were collected at three different times: prior to surgery (preOP), on the first postoperative day (POD1) and about six weeks after PD during the first medical follow-up (POW6). Samples used for the stimulation and flow cytometry assays were collected into heparin containing tubes (S-Monovette, Sarstedt AG, Nümbrecht, Germany) and processed immediately within two hours after collection. EDTA-containing tubes were used to collect plasma for the proteomic analysis and stored at −80 °C until further processing.

### Whole blood stimulation

Whole blood cells were diluted 1:10 with RPMI cell culture medium (Thermo Fisher Scientific, Waltham, MA, USA) containing 1% Penicillin/Streptavidin. Stimulation was done in duplicate, either with the superantigen SEB (Staphylococcal enterotoxin B, Sigma Aldrich, St. Louis, MO, USA) with a final concentration of 0.5 µg/mL or with LPS (Lipopolysaccharide, Sigma Aldrich, St. Louis, MO, USA) with a final concentration of 0.01 µg/mL. The stimulated samples were incubated in 24-well plates for 24 h at 37 °C in a 5% CO_2_ atmosphere. Thereafter, the samples were centrifuged and the supernatant was harvested and stored in aliquots at −80 °C for further analysis, while the pellets were rediluted in 100 µL PBS (Lonza Group, Basel, CH) and stained for flow cytometry analysis (see flow cytometry).

### Enzyme-linked immunosorbent assay (ELISA)

Cytokines were quantified in the supernatant after stimulation (IFNγ, IL2, IL10, TNFα) or in the unstimulated plasma (IL6) with ELISA-technique using DuoSet ELISA-Kits (R&D Systems, Abingdon, UK) following manufacturer-provided instructions.

### Flow cytometry

For the cell staining of 100 µL of whole blood or of 100 µL of the diluted pellet, they were incubated for 20 min with the following mixtures of fluorescent monoclonal surface marker specific antibodies: (1) anti-CD4-FITC/anti-CD25-PE/anti-CD3-PerCP-Cy 5.5 for T-cell subsets and activated T-cells, (2) anti-CD14-PE/anti-CD16-FITC for monocyte subsets [22], (3) anti-CD4-FITC/anti-CD25-PE-Cy7/anti-CD127-Alexa Fluor 647 for Tregs [23]; and (4) anti-CD4-FITC/anti-CD69-PE/anti-CD3-PerCP-Cy 5.5 for activated T-cells. For leukocyte fixation and red blood cell lysis, 100 µL of Optilyse B (Beckman Coulter, Brea, CA, USA) was added, followed by 1 mL of Aqua dest. Single cells were measured with a flow cytometer (BD LSR Fortessa, BD Biosciences, Franklin Lakes, NJ, USA). Flow cytometry data was analyzed with the FlowJo V10 software determining main cell populations in forward and sideward scatter, followed by the identification of the further specific subsets based on the fluorescence-stained surface markers.

### Proteomic analysis

To compare perioperative plasma protein expression profiles between the two groups, we used the Proximity Extension Assay (PEA) technology. In summary, specific antibody pairs labelled with unique oligonucleotides bind to the target proteins in the sample. In case of a pairwise binding to the target and the oligonucleotides being in close proximity, short nucleotide sequences are formed by hybridization and polymerization processes. The resulting nucleotide sequences serve as markers for the target proteins and are then quantified through realtime-PCR. Avoiding cross-reactive events using paired oligonucleotides, this technique allows the simultaneous detection of many more proteins than conventional immunoassays without a loss of specificity [24].

PEA was performed by Olink Proteomics (Uppsala, Sweden) using the multiplex panel ONCOLOGY II, which includes 92 cancer-related proteins. The samples of the patients with benign diseases were analyzed separately, while the samples of cancer patients were pooled into seven samples to display the protein profile at the time points preOP and POW6. The resulting data was given in the Olink own arbitrary unit, Normalized Protein eXpression (NPX), which is in binary logarithm. For further statistical analysis, it was converted into linear scale.

### Statistical analysis

Data analysis was done using IBM SPSS statistics software (Version 23.0, IBM Corporation, Armonk, NY, USA). Before further comparative analysis, the Shapiro–Wilk test was carried out, assessing whether our data follows normal distribution. Categorical variables were compared using Fisher’s exact test (frequencies of less than five in a group), and the comparison of continuous variables was performed using the unpaired Student’s *T*-test (in case of normal distribution) or the Mann–Whitney-*U*-test as a non-parametric test. To test for differences between the time points within one group, we did a paired T-test, or in the case of more than two time points, a repeated measures ANOVA (analysis of variance) and the Bonferroni-corrected post-hoc test or the Friedmann test as a non-parametric alternative. Receiver operating characteristic (ROC) curves and corresponding area under the curve (AUC) values were performed to define appropriate cut-off-values regarding the immunological parameters followed by a comparative analysis using Kaplan–Meier curves and the log-rank test. Any p-value < 0.05 was considered to be statistically significant (*p < 0.05; **p < 0.01; ***p < 0.001).

## Results

### Patient characteristics

38 patients with the indication for pancreatoduodenectomy were included, among them seven who had been excluded subsequently due to severe early postoperative complications interfering with the natural course after PD (reoperation due to postoperative bleeding n = 4 or anastomotic insufficiency (n = 2) and severe postoperative infection with sepsis n = 1). According to the Clavien Dindo classification, three of these complications could be classified to IIIb, two to grade IVa and two to grade V. Corresponding to the final histopathology, 24 patients were assigned to the adenocarcinoma group (adenocarcinoma of the pancreatic head and its periampullary region) and seven patients to the control group (benign pancreatic diseases). Clinical and demographic characteristics are listed in Tables 1 and 2. The operative time was significantly longer for adenocarcinoma patients. However, regarding the distribution of the patients’ baseline characteristics and the further perioperative clinical data, no significant differences were observed between the two groups. None of the patients received neoadjuvant or adjuvant chemotherapy during the period of sample collection.

**Table 1:** Demographic and clinical data

	Adenocarcinoma (n = 24)	Benign (n = 7)	Total (n = 31)	p-value
Age mean, years (range)	69.5 (46–84)	68.7 (50–79)	69.3 (46–84)	0.68^a^
Sex, n (%)				
Male	17 (70.8)	5 (71.4)	22 (71.0)	0.85^b^
Female	7 (29.2)	2 (28.6)	9 (29.1)	
Surgery, n (%)				
Open	11 (45.8)	4 (57.1)	15 (48.4)	1.0^b^
Laparoscopy-assisted	11 (45.8)	3 (42.9)	14 (45.2)	
Conversion	2 (8.3)	–	2 (6.5)	
Histopathological diagnosis, n				
PDAC	21		21	
Distal Cholangio-carcinoma	2		2	
Duodenal cancer	1		1	
IPMN		4	4	
Cystadenoma		2	2	
PanIN 1A		1	1	
Blood loss mean, ml (range)	881.25 (200–2900)	450.0 (200–850)	783.87 (200–2900)	0.51^c^
Operative time mean, min (range)	468.38 (245–612)	349.57 (235–482)	441.55 (235–612)	0.01^a^
ICU stay mean, days (range)	5.6 (3–29)	4.1 (3–6)	5.3 (3–29)	0.7^c^
Overall hospital stay, days (range)	22.9 (9–62)	16.4 (13–22)	21.4 (9–62)	0.36^c^
Survival, n				
Died	18			
Alive	4			
Lost to follow up	2			

n: number of patients, PanIN: Pancreatic intraepithelial neoplasia, IPMN: Intraductal papillary mucinous neoplasm, PDAC: Pancreatic ductal adenocarcinoma, ICU: intensive care unit

^a^Unpaired Student’s T-test

^b^Fisher’s exact test

^c^Mann–Whitney-*U*-test

**Table 2:** Tumor classification

Pathologic disease stage, n (%)		Grading, n (%)		UICC stage, n (%)		Resection margin status, n (%)	
pT2	1 (4.2)	G2	7 (29.2)	IIA	4 (16.7)	R0	21 (87.5)
pT3	22 (91.7)	G3	17 (70.8)	IIB	19 (79.2)	R1	3 (12.5)
pT4	1 (4.2)			III	1 (4.2)		
pN0	4 (16.7)						
pN1	20 (83.3)						

n: number of patients, pT: pathological tumor stage (size and local invasion), pN: pathological lymph nodes stage (involvement of regional lymph nodes), UICC: Union internationale contre le cancer

### Immune status prior to surgery (preOP)

#### Peripheral white blood cell populations

**Fig. 1:**
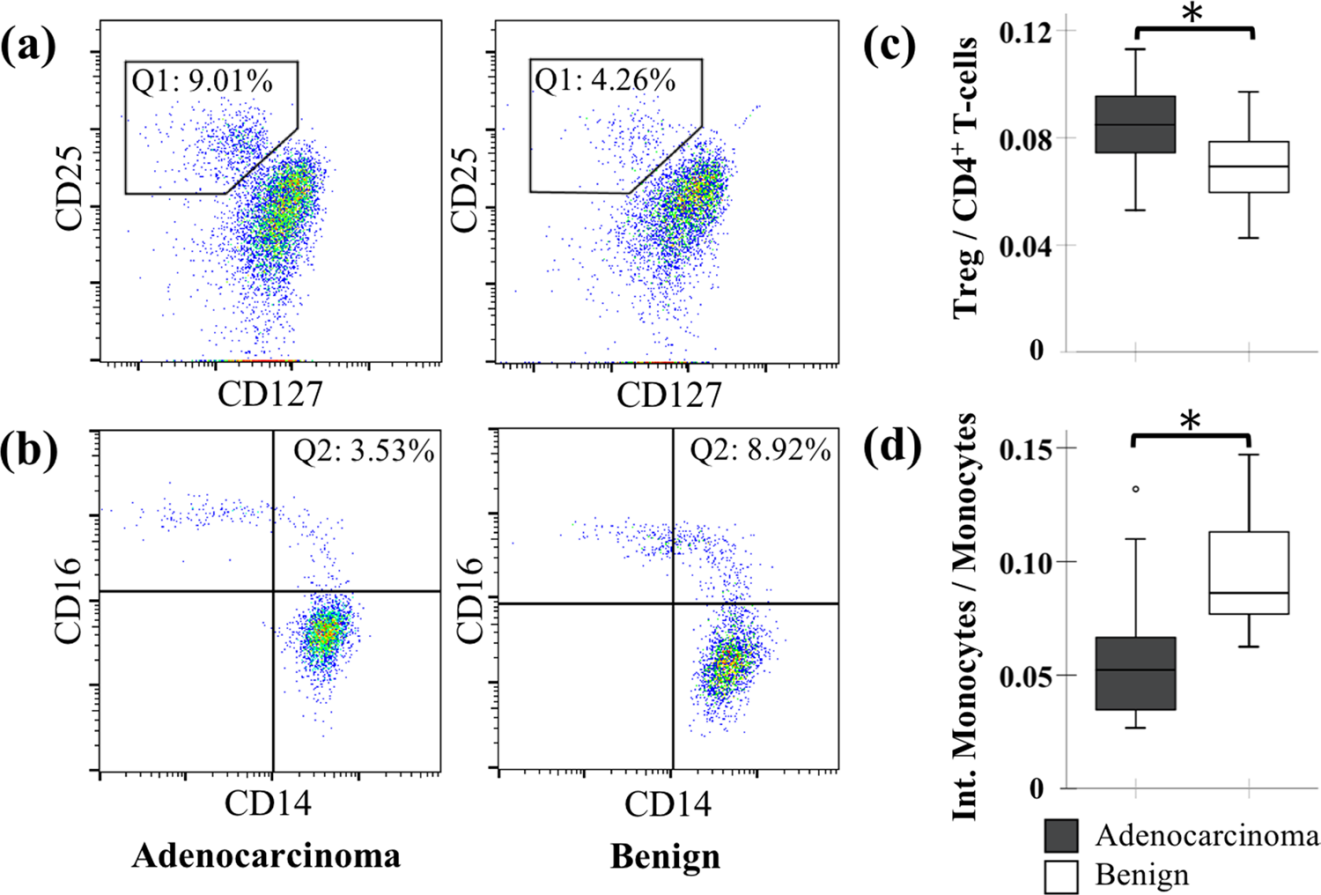
Preoperative differences in peripheral Tregs and intermediate monocytes. Representative flow cytometry dotplots of an adenocarcinoma sample (left side) and a benign pancreatic disease sample (right side) showing **a** the percentage of Tregs (Q1: CD4^+^/CD25^+^/CD127^low^) of total CD4^+^ T-cells and **b** the percentage of intermediate monocytes (Q2: CD14^++^/CD16^+^) of total monocytes. Corresponding boxplots showing quantification data: **c** the mean proportion of Tregs relative to the total CD4^+^ T-cells and **d** the mean proportion of intermediate monocytes relative to the monocyte population. The boxplots represent the distribution of the data with the box showing the interquartile range, the horizontal line showing the median, the whiskers showing the 1.5 IQR value and circles showing outliers. *p < 0.05 (unpaired Student’s *T*-test)

To begin with, we did not find any significant difference in the total leucocytes, lymphocytes or monocytes in preoperative blood samples between the two groups. The flow cytometric immune phenotyping revealed significantly higher levels of Tregs in adenocarcinoma patients (Fig. 1a, c) but no difference in the expression of the lymphocytic surface markers CD3 or CD4 (Additional file 1: Table S1). Moreover, we found a significantly lower proportion of CD14^++^/CD16^+^ intermediate monocytes in patients with adenocarcinoma before the operation (Fig. 1b, d), which are commonly defined as a proinflammatory monocyte subset.

#### Expression of immune-associated and inflammatory proteins

**Fig. 2:**
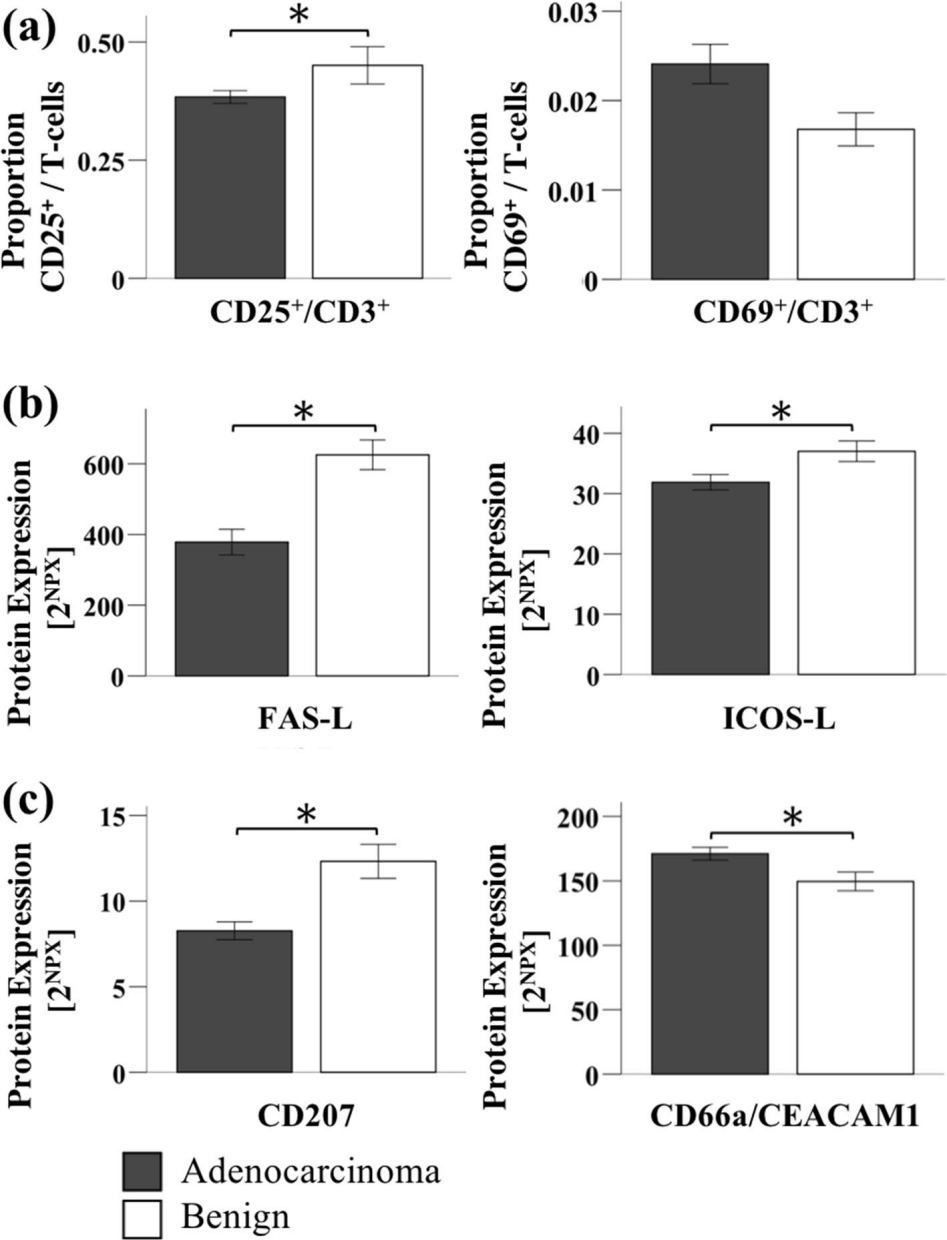
Identification of preoperative immune-associated proteins. Selected preoperative data is shown comparing **a** the surface activation markers on lymphocytes (flow cytometry data), lymphocyte-associated **b** ligands and **c** other protein biomarkers (data from proteomic analysis) between patients with adenocarcinoma and patients with benign pancreatic diseases. Bar charts and error bars represent mean ± SEM. *p < 0.05 (unpaired Student’s *T*-test)

We next analyzed immune-associated inflammatory proteins. Regarding T-cell activation in vivo, we found a significantly lower expression of the activation marker CD25 on total CD3^+^ T cells in the adenocarcinoma patients, whereas no differences were found in the expression of CD69 (Fig. 2a). When we performed the PEA, we found decreased levels of the two lymphocyte-binding ligands FAS-L and ICOS-L in the plasma of adenocarcinoma patients (Fig. 2b). We also found a significantly different expression of two very important plasma proteins related to lymphocyte functions: CD207, which showed a decreased plasma concentration, and CD66a/CEACAM1, which showed higher plasma levels in the adenocarcinoma group (Fig. 2c). Further biomarkers, generally associated with inflammatory processes, such as CYR61, TRAIL, MADH5 (a member of the TGF-ß superfamily) and MIC-A/B (MHC class I polypeptide-related sequences A/B) showed different preoperative plasma levels when comparing the two groups. The plasma level of IL6, a classical acute-phase mediator, did not differ between patients with cancer and benign pancreatic disease (Additional file 1: Table S1).

#### Whole blood stimulation

**Fig. 3:**
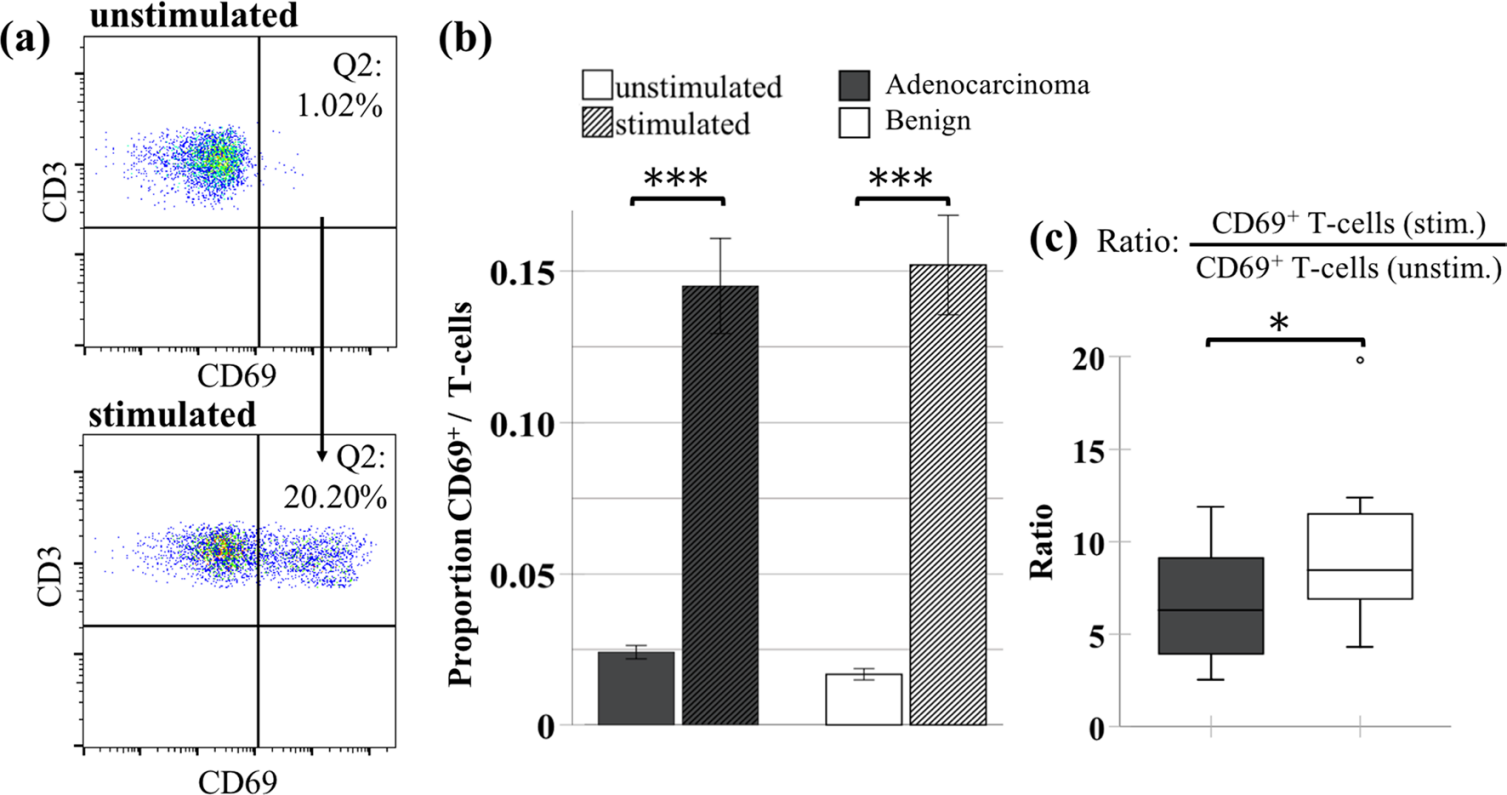
Comparison of CD69 upregulation in vitro after SEB stimulation. **a** Representative flow cytometry dotplots of the same patient showing the percentage of CD69^+^/ CD3^+^ T-cells among the total CD3^+^ population (Q2: CD3^+^/CD69^+^) before and after in vitro stimulation with SEB. Corresponding summary data is depicted as **b** bar charts with error bars showing the mean proportion ± SEM of CD69^+^ T-cells (relative to the total T-cells) before and after in vitro stimulation with SEB grouped by underlying diseases and **c** boxplots comparing the stimulation ratio (between the proportion of CD69^+^ T-cells in the stimulated sample and in the unstimulated sample). The boxplots represent the distribution of the data with the box showing the interquartile range, the horizontal line showing the median, the whiskers showing the 1.5 IQR value and circles showing outliers. *p < 0.05, ***p < 0.001 (**b**: paired *T*-test, **c** unpaired Student’s *T*-test)

Upon T-cell stimulation with SEB, both groups showed a significant increase in peripheral CD69 expressing CD3^+^ T-cells. We observed the same effect in the CD4^±^ subsets (Fig. 3a/b, Additional file 1: Table S2). To improve the comparison, we calculated the ratio between the proportion of CD69^+^ T-cells in the stimulated sample and in the unstimulated sample. The ratio was significantly decreased in adenocarcinoma patients, indicating a reduced lymphocytic activation by SEB (Fig. 3c). Contrary to CD69 upregulation, the stimulation with SEB had no significant effect on the expression of the activation marker CD25 after 24 h of stimulation. When we analyzed the cytokine release by T cells after SEB stimulation, the concentration of IL2 and IFNγ in the supernatant of the cell cultures was below the detection limit in unstimulated cell cultures. But in response to the stimulation with SEB, we noticed a potent cytokine release in both groups, while the presence of an adenocarcinoma did not have a significant influence (Additional file 1: Table S1). Analogous to this experiment, we used LPS as a stimulating agent for monocytes and assessed the TNFα and IL10 release. The unstimulated group had low levels of TNFα and IL10, however, upon stimulation, we saw an additional in vitro release of TNFα and IL10 in both groups, but the release was not different in samples of patients with adenocarcinoma.

#### Early postoperative immune and inflammatory response (POD1)

**Fig. 4:**
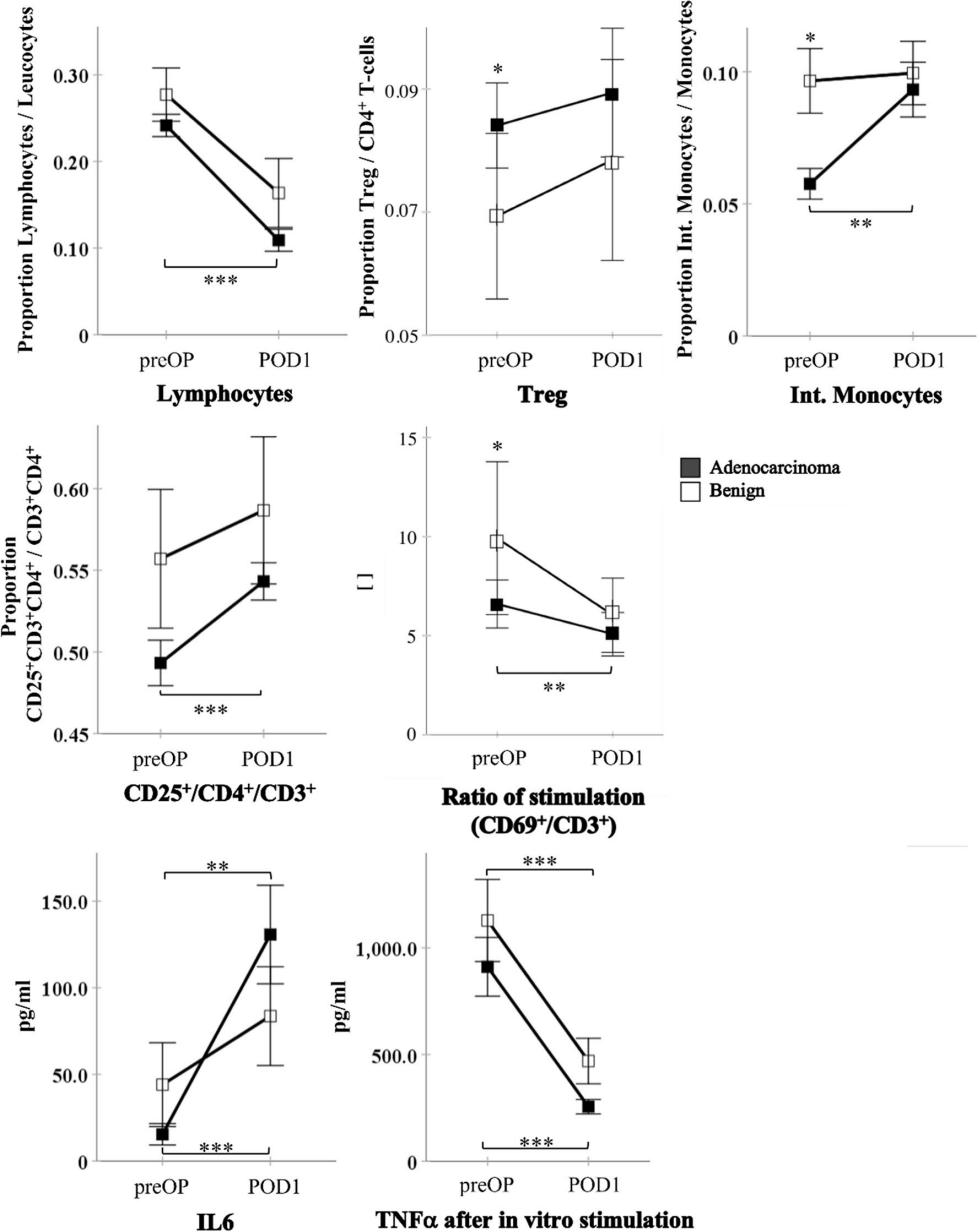
Kinetics of immunologic parameters in the short-term perioperative course (first postoperative day). Selected preoperative (preOP) and postoperative (POD1) results are shown, comparing between patients with adenocarcinoma and patients with benign pancreatic diseases at each time point and showing the perioperative changes. Data points show mean ± SEM. *p < 0.05, **p < 0.01; ***p < 0.001 (Lymphocytes, Tregs, CD25^+^/CD4^+^/CD3^+^, Ratio of stimulation, TNFα/benign: ANOVA; Int. Monocytes, IL6, TNFα/adenocarcinoma: Friedmann test)

On the first postoperative day, the proportion of peripheral blood lymphocytes decreased significantly in both groups without any differences between the groups (Fig. 4). Monocytes and CD3^+^ T-cells remained unaltered in both groups, but in both groups the PD induced a significant elevation of the CD4^−^ proportion, while the CD4^+^ proportion was reduced when compared to the preoperative baseline (Additional file 1: Table S1). As we have preoperatively seen an elevated level of Tregs in adenocarcinoma patients, we now saw a Treg population that was no longer different from that of the patients with benign diseases (Fig. 4). The initial cancer-associated reduction of intermediate monocytes also reached comparable levels to non-cancer patients (Fig. 4). Regarding the activation level of circulating T-cells in vivo, the portion of activated CD25^+^ CD4^+^ helper T-cells showed a significant increase in cancer patients on the first postoperative day (Fig. 4), while the CD25 expressing overall CD3^+^ cells and CD4^−^ subset did not change significantly in the perioperative course (Additional file 1: Table S1).

When we compared the expression of CD25 on CD3^+^ T-cells between the two groups, which was reduced before the PD in adenocarcinoma patients, we did not notice any difference postoperatively.

The in vitro T-cell stimulation showed a reduced stimulability in both groups on POD1 when compared to the preoperative baseline (reduced ratio between the proportion of CD69^+^ T-cells in the stimulated sample and in the unstimulated sample). Here, the perioperative decrease of T-cell activation capability reached significance within the adenocarcinoma group. The comparison between the groups on POD1 showed no significant difference anymore (Fig. 4).

Stimulated IL2, IFNγ, and IL10 release were not affected by the PD, but the release of TNFα after stimulation with LPS was significantly reduced on the first postoperative day in both groups (TNFα: Fig. 4, other cytokines: Additional file 1: Table S1). The serum concentration of IL6 reached peak levels in both groups, with an eight-fold increase observed in cancer patients when compared to the preoperative level and twice increased concentrations in patients with benign pancreatic diseases (Fig. 4).

#### Long-term changes after surgical resection (POW6)

**Fig. 5:**
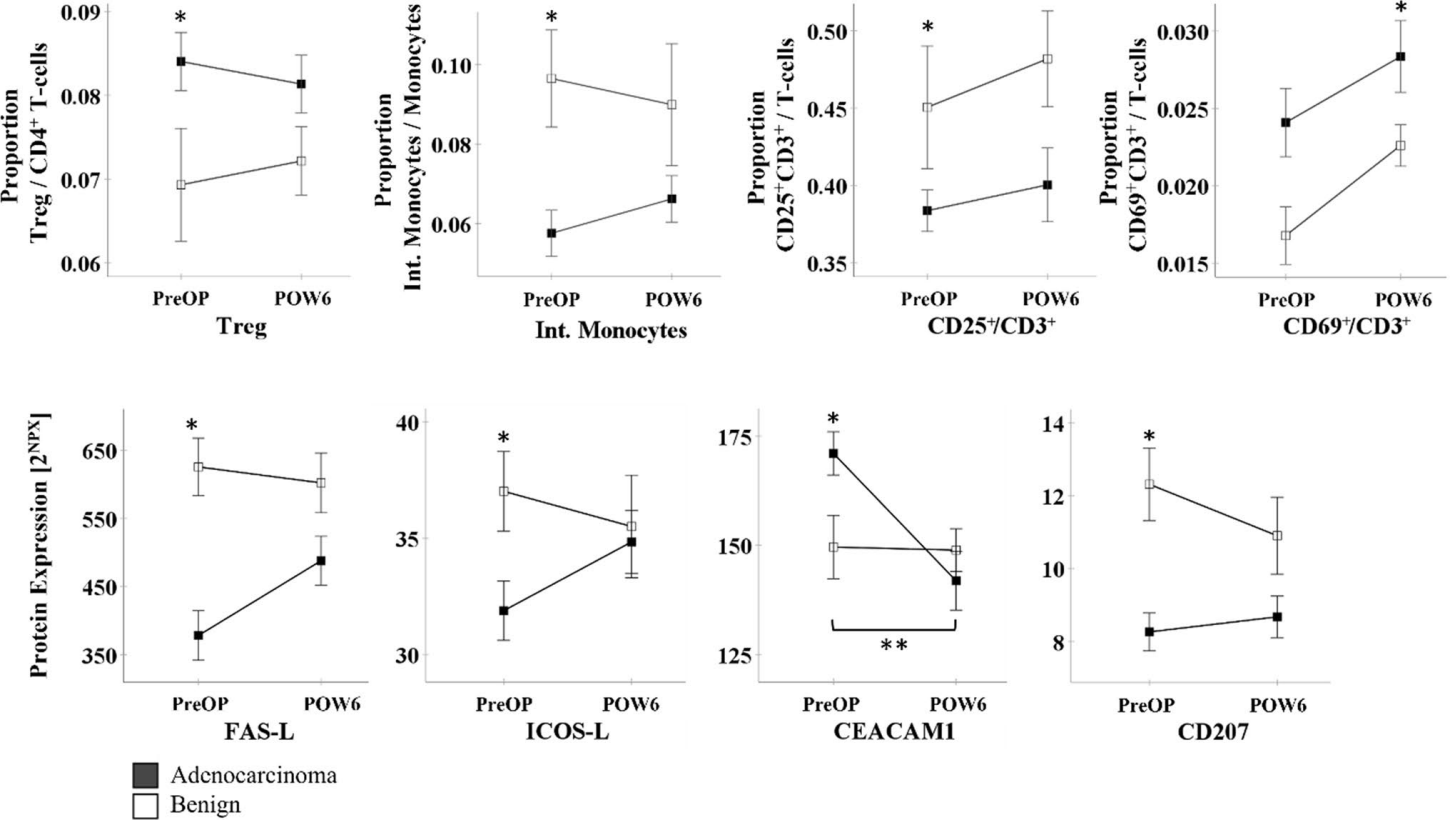
Kinetics of immunologic parameters in the longer-term perioperative course (six weeks after PD). Selected preoperative (preOP) and postoperative (POW6) results are shown, comparing between patients with adenocarcinoma and patients with benign pancreatic diseases at each time point and showing the perioperative changes. Data points show mean. *p < 0.05 (Tregs, CD25^+^/CD3^+^/benign, CD69^+^/CD3^+^: ANOVA; Int. Monocytes, CD25^+^/CD3^+^/adenocarcinoma: Friedman test; FAS-L, ICOS-L, CEACAM1, CD207: paired T-test)

Approximately six weeks after PD, none of the preoperatively observed differences between cancer and non-cancer patients remained detectable (Fig. 5). Thus, the analysis of the cellular parameters (Tregs, intermediate monocytes), plasma proteins (CD25 on CD3^+^ T-cells, FAS-L, ICOS-L, CEACAM1/CD66a, CD207, CYR61, MADH5, MIC-A/B), and functional assays (in vitro stimulation of CD69-expression and cytokine release) did not provide evidence for persistent alterations of the immune status in adenocarcinoma patients (Fig. 5, Additional file 1: Table S1). However, six weeks after PD, the proportion of peripheral CD3^+^ lymphocytes expressing the activation marker CD69 was significantly elevated in cancer patients, which we did not see prior to surgery (Fig. 5). This suggests a partial recovery of the immune system in patients with neoplastic disease after removal of the malignant tumor.

#### Follow-up and prognostic impact

After enrollment of the last patient, the adenocarcinoma patients were followed up for 36 months. In total, 18 patients with adenocarcinoma have died during the period of follow-up, which included all patients with R1-resection. Two patients failed to follow up so their survival data was unavailable. The median postoperative survival time was 17 months.

**Fig. 6:**
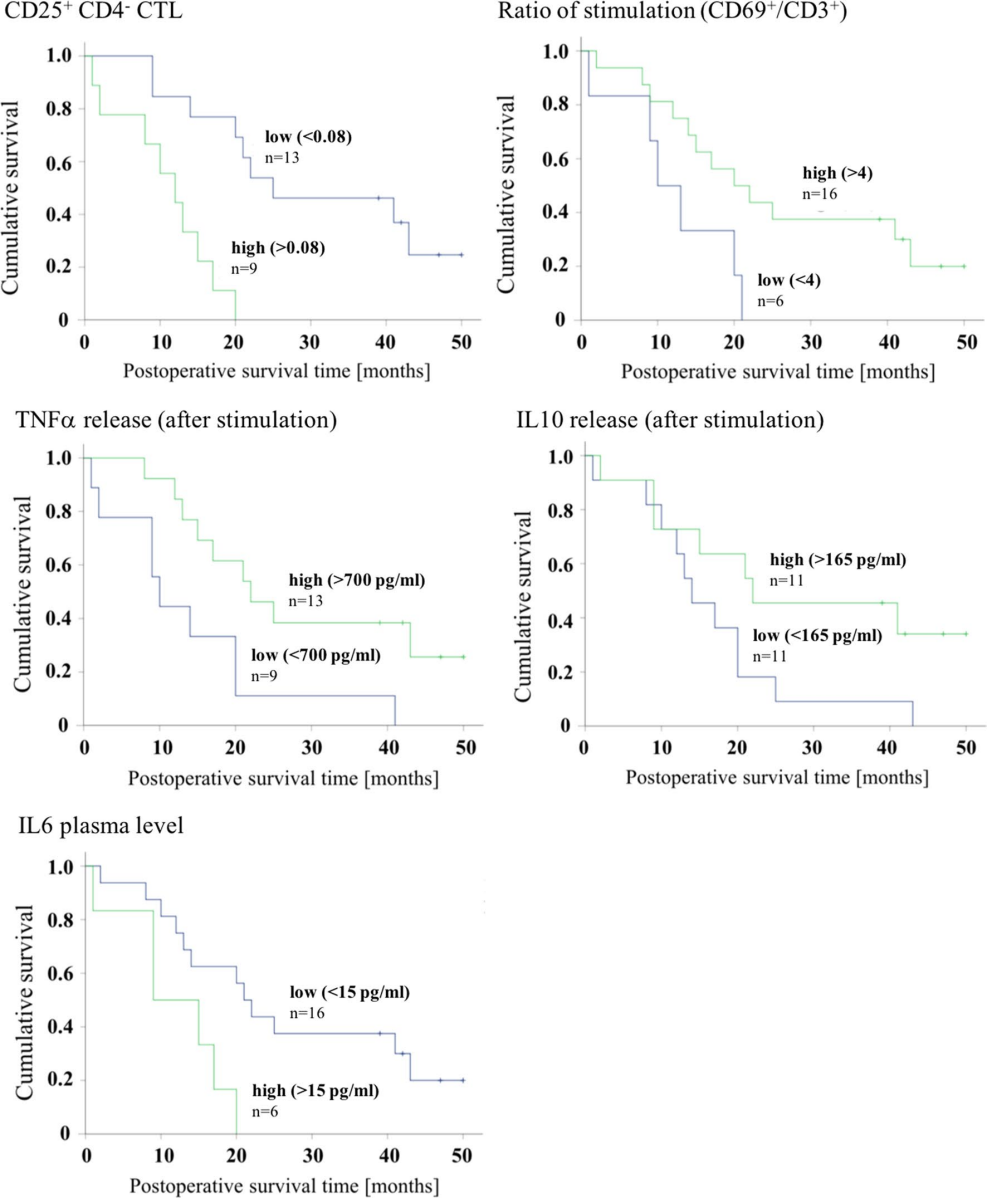
Kaplan–Meier analysis of postoperative survival in adenocarcinoma patients regarding immunologic parameters. Grouping of the patients by high and low levels of the parameters is based on thresholds deriving from the ROC curves. Differences in survival between the groups were calculated with the log-rank test: CD25^+^/CD4^−^ CTL: p < 0.001, Ratio of stimulation (CD69^+^/CD3^+^): p = 0.033, TNFα release: p = 0.013, IL10 release: p = 0.057 and IL6 plasma level: p = 0.018

To evaluate the prognostic relevance of the immunological parameters, the survival of adenocarcinoma patients was analyzed with respect to the preoperative immune function. Based on the thresholds derived from the ROC-curves, the patients were grouped by high and low levels of the given parameters and compared with Kaplan–Meier survival analysis and log-rank test, which is represented in Fig. 6. The postoperative survival time was significantly reduced in patients with high levels of activated cytotoxic T-cells (CD4^−^/CD25^+^), (25 months vs. 12 months, log-rank test p < 0.001), in patients with a decreased CD69 stimulation ratio (20 months vs. 10 months, log-rank test p = 0.033), in patients with elevated plasma levels of IL6 (21 months vs. 9 months; log-rank test p = 0.018) and in patients that showed low release of TNFα after in vitro stimulation (22 months vs. 10 months; log-rank test p = 0.013). High IL10 release after stimulation with LPS was associated with increased survival of 22 months in patients with high release vs. 14 months in patients with low release (log-rank test p = 0.057).

## Discussion

While the presence of a periampullary adenocarcinoma appears to have no impact on the immediate surgery-induced postoperative immune and inflammatory reaction, we demonstrate here, that these patients show systemic immunological alterations preoperatively that are reversible by removing the tumor. Moreover, the influence of an adenocarcinoma on the immune system is correlated with the postoperative survival, suggesting that pre- and postoperative samples may be an excellent cohort to identify relevant changes of the immune system by pancreatic and periampullary malignancies undergoing PD.

The systemic tumor-associated impairment of the host immune system has been described by many previous studies [7–9]. In contrast to these reports, we describe the immunological dynamics in the perioperative period comparing patients with adenocarcinoma to non-cancer patients. One of our major preoperative findings are alterations in the population of intermediate monocytes. Beside the predominant group of CD14^++^/CD16^−^ classical monocytes and the CD14^+^/CD16^++^ non-classical monocytes, the intermediate monocytes show high levels of CD14 together with low CD16 expression (CD14^++^/CD16^+^) [22]. Commonly they are linked to inflammation as they are shown to produce proinflammatory cytokines and an expansion has been described in inflammatory diseases [25–27]. Although cancer is closely associated with inflammation, little is known about their role in cancer so far. Elevated numbers of intermediate monocytes have been reported in colorectal cancer [28], but in our cohort, a decreased proportion was detected in the peripheral blood of patients with adenocarcinoma. Thus, further trials are required to verify our results and to examine their specific role in cancer, especially in view of cancer- and surgery-induced inflammation. The malignant disease further induced changes in Treg populations, a subset of CD4^+^ T-lymphocytes. They are characterized by their immunoregulatory activity and are deemed to play a central role inhibiting T-effector cells and releasing immunosuppressive cytokines. Increased levels of Tregs are described at the tumor site as well as in the peripheral blood, with high numbers of Tregs being associated with tumor progression and poor prognosis in many tumor entities including PC [7, 29, 30]. In line with these findings, we detected a higher frequency of Tregs in the peripheral blood of cancer patients, which could be an indication for a systemic tumor-associated immunosuppression. Our findings regarding the lymphocyte activation markers CD25 and CD69 in vivo and in stimulated whole blood cultures would support the idea of an impaired immune system in adenocarcinoma patients in a functional context. However, the release of cytokines in response to in vitro stimulation was comparable in both groups, suggesting that this part of the effector function has been maintained. Together, our preoperative data provides evidence for systemic immunologic changes in cancer patients, but as our study only covers a small part of the immunologic landscape and the adenocarcinoma group includes heterogenous entities, our results do not permit a definite interpretation of the systemic immunological conditions.

Considering the perioperative dynamics, on the first postoperative day we observed systemic changes regardless of the underlying disease, e.g. a shift in the distribution of CD4^±^ cells and a decreased release of TNFα after stimulation in vitro when compared with preoperative levels. These results could be interpreted as an inflammatory response due to the surgical trauma concerning both groups, whose mechanisms can be classified within the concepts of SIRS and CARS leading to postoperative immunosuppression and to increased susceptibility to infectious complications [14, 15, 31]. Our results correspond to studies on patients with severe trauma, which report an altered composition of circulating immune cells and a decreased release of cytokines [32, 33]. This postoperative immunosuppression is a potential target to improve the prognosis of patients undergoing tumor resection, on the one hand to generally reduce postoperative infections and on the other hand to rapidly restore the anti-tumor immunity to minimize the risk of tumor recurrence or metastasis. As an example, reducing the surgical invasiveness using laparoscopic procedures was associated with an improved long-term survival and a maintenance of the postoperative immunity in colorectal cancer patients [18, 34, 35]. Furthermore, combining surgery and medical immune modulation would be another promising strategy [36]. Regarding the benign pancreatic diseases in our study, which are considered as precursor lesions to pancreatic cancer, it can be assumed, that a well-maintained immunosurveillance might be essential for disease control and an impairment due to postoperative SIRS and CARS might promote a recurrence even in benign tumors.

After the initial inflammatory reaction, the preoperative immunological alterations associated with the tumor could no longer be proven when assessed six weeks after the operation. This is in accordance to previous studies demonstrating a postoperative restoration of the immune function in solid cancer patients and in murine models [7, 37]. Consequently, after a temporary immunosuppression, surgical therapy seems to contribute to the recovery of the protective immune response. In line with our results, there is evidence in patients with resected esophageal or gastroesophageal junction cancer, that initiating immunotherapy at least 10 weeks after the operation improves disease-free survival [38]. Here, the temporary postoperative immunosuppression shown in our study could be an underlying mechanism.

Interestingly, the proportion of circulating activated CD69^+^ T-cells was elevated in cancer patients, which we did not see before the operation. Persistent alterations of the immune status could be an early indicator for remaining tumor cells or metastases that continue modulating the immune system. This could help to identify patients at risk for local recurrence or metastasis. Appropriately, postoperative inflammatory parameters were demonstrated in colorectal cancer patients to predict the overall and recurrence-free survival [39, 40]. However in our study, because our preoperative data in adenocarcinoma patients does not provide a value that represents the baseline and number of patients is small, a postoperative differentiation based on individual values would be difficult.

Moreover, the preoperative immune profile in adenocarcinoma patients correlated with the postoperative survival. This is consistent to previous studies in several tumor types including PC, that also showed the prognostic relevance of immunologic parameters [7, 10, 41]. These parameters might serve as prognostic biomarkers and as potential targets for treatment strategies depending on individual risk factors. Regarding the postoperative follow-up, the mentioned parameters might also be potential biomarkers in order to find residual disease or recurrence, what needs to be proven in further studies.

Finally, the main limitations of our study are the small size of included subjects, the heterogeneity within the groups and an unbalanced number of cancer patients and patients in the control group. Here, it must be emphasized that the control group contains patients with benign pancreatic diseases including PC precursor lesions, which are suspected to already bear immunologic abnormalities [42]. Thus, while our findings are difficult to generalize, they nonetheless implicate potential prognostic and therapeutic possibilities in patients with resectable periampullary cancer. Therefore, larger comparative studies, especially with healthy controls, should verify our preoperative findings.

## Conclusions

In summary, we demonstrate systemic immunological alterations in patients with resectable cancer that are reversible after tumor resection, while the immediate postoperative immune reaction seems to be unaltered. Moreover, the preoperative immune status affects the postoperative survival. Our study provides new insight into the perioperative period in patients with resectable cancer and implies that pre- and postoperative samples may be a key object of study to identify relevant prognostic and therapeutic targets. To translate our findings to clinical practice and especially to further involve immunotherapy in curative therapeutical regimes and to validate pre- and postoperative prognostic biomarkers, more detailed studies are required to determine the underlying mechanisms on how anti-tumor immunity changes during tumor growth and in the perioperative course.

### Supplementary Information

**Additional file 1: Table S1.** Complete listing of all parameters analyzed (preoperatively = preOP, on post-OP day 1 = POD1 and approximately six weeks after surgery = POW6) and comparison between the groups at one point in time and within the groups over time; Table S2: Effect of whole blood stimulation with SEB on the expression of CD69 on CD3^+^, CD3^+^/CD4^+^ and CD3^+^/CD4^−^ lymphocytes.

## Data Availability

The datasets generated during and/or analysed during the current study are available from the corresponding author on reasonable request.
