# Supplementary material for: Immune changes induced by periampullary adenocarcinoma are reversed after tumor resection and modulate the postoperative survival
Source: Discov Oncol. 2023 Aug 23;14:153. doi: 10.1007/s12672-023-00768-2 (PMC10447764; doi:10.1007/s12672-023-00768-2)
Supplement: Supplementary file 1 — Additional file 1: Table S1. Complete listing of all parameters analyzed (preoperatively = preOP, on post-OP day 1 = POD1 and approximately six weeks after surgery = POW6) and comparison between the groups at one point in time and within the groups over time; Table S2: Effect of whole blood stimulation with SEB on the expression of CD69 on CD3+, CD3+/CD4+ and CD3+/CD4− lymphocytes. [file 12672_2023_768_MOESM1_ESM.docx]

***Supplementary Materials***

**Table S1**. Complete listing of all parameters analyzed (preoperatively=preOP, on post-OP day 1= POD1 and approximately six weeks after surgery= POW6) and comparison between the groups at one point in time and within the groups over time.

|  | **Adenocarcinoma** | **Benign** | **p-value^a^** |
| --- | --- | --- | --- |
|  | *[10^3/µl] (means ± SEM)* | |  |
| Leukocytes   - PreOP | 7.600 ± 0.440 | 8.106 ± 1.067 | 0.562 |
|  | *[10^3/µl] (means ± SEM)* | |  |
| Lymphocytes   - PreOP | 0.242 ± 0,0129 | 0.277 ± 0,0308 | 0.228 |
| - POD1 | 0.109 ± 0.0127 ^sig0^ | 0.164 ± 0.0398 | 0.199^b^ |
| - POW6 | 0.246 ± 0.0167 ^sig1^ | 0.289 ± 0.0338 | 0.221 |
| Monocytes   - PreOP | 0.062 ± 0.0048 | 0.056 ± 0.0099 | 0.542 |
| - POD1 | 0.057 ± 0.0041 | 0.055 ± 0.0077 | 0.802 |
| - POW6 | 0.065 ± 0.0063 | 0.058 ± 0.0054 | 0.394 |
| T-Lymphocytes (CD3^+^)   - PreOP | 0.657 ± 0.0157 | 0.595 ± 0.0376 | 0.092 |
| - POD1 | 0.599 ± 0.0249 | 0.494 ± 0.0643 | 0.078 |
| - POW6 | 0.662 ± 0.0176 | 0.644 ± 0.0257 ^sig1(c)^ | 0.603 |
| TH-L (CD3^+^/CD4^+^)   - PreOP | 0.706 ± 0.0240 | 0.754 ± 0.0428 | 0.341 |
| - POD1 | 0.668 ± 0.0255 ^sig0^ | 0.702 ± 0.0420 ^sig0^ | 0.522 |
| - POW6 | 0.699 ± 0.0321 | 0.744 ± 0.0395 ^sig1^ | 0.459 |
| CT-L (CD3^+^/CD4^-^)   - PreOP | 0.294 ± 0.0240 | 0.246 ± 0.0428 | 0.341 |
| - POD1 | 0.332 ± 0.0255 ^sig0^ | 0.298 ± 0.0420 ^sig0^ | 0.522 |
| - POW6 | 0.300 ± 0.0322 | 0.256 ± 0.0395 ^sig1^ | 0.459 |
| Tregs  (CD4^+^/CD25^++^/CD127^low^)   - PreOP | 0.084 ± 0.0035 | 0.069 ± 0,0067 | 0.047 |
| - POD1 | 0.089 ± 0.0052 | 0.078 ± 0.0082 | 0.289 |
| - POW6 | 0.081 ± 0.0034 | 0.072 ± 0.0041 | 0.128 |
| Intermediate monocytes  (CD14^++^/CD16^+^)   - PreOP | 0.058 ± 0.0058 | 0.097 ± 0.0122 | 0.004 |
| - POD1 | 0.093 ± 0,0104 ^sig0(c)^ | 0,099 ± 0.0120 | 0.872^b^ |
| - POW6 | 0.066 ± 0,0059 | 0,090 ± 0.0153 | 0.086 |
| Activated CD25^+^/CD3^+^   - PreOP | 0.384 ± 0.0134 | 0.451 ± 0.0396 | 0.048 |
| - POD1 | 0.412 ± 0.0203 | 0.453 ± 0.0418 | 0.357 |
| - POW6 | 0.400 ± 0.0239 | 0.482 ± 0.0309 | 0.075 |
| Activated CD25^+^/CD3^+^/CD4^+^   - PreOP | 0.493 ± 0.0139 | 0.557 ± 0.0426 | 0.072 |
| - POD1 | 0.543 ± 0.0115 ^sig0^ | 0.587 ± 0.0451 | 0.381 |
| - POW6 | 0.521 ± 0.0201 ^sig0^ | 0.603 ± 0.0371 | 0.051 |
| Activated CD25^+^/CD3^+^/CD4^-^   - PreOP | 0.128 ± 0.0217 | 0.147 ± 0.0715 | 0,695 ^b^ |
| - POD1 | 0.127 ± 0.0212 | 0.157 ± 0.0767 | 0,800 ^b^ |
| - POW6 | 0.105 ± 0.0179 | 0.155 ± 0.0640 | 0,821 ^b^ |
| Activated CD69^+^/CD3^+^   - PreOP | 0.024 ± 0.0022 | 0.017 ± 0.0019 | 0.096 |
| - POD1 | 0.024 ± 0.0024 | 0.021 ± 0.0025 | 0.467 |
| - POW6 | 0.028 ± 0.0023 | 0.023 ± 0.0013 | 0.042 |
| Activated CD69^+^/CD3^+^/CD4^+^   - PreOP | 0.012 ± 0.0013 | 0.010 ± 0.0025 | 0.182 ^b^ |
| - POD1 | 0.013 ± 0.0017 | 0.012 ± 0.0024 | 0.886 ^b^ |
| - POW6 | 0.013 ± 0.0009 | 0.012 ± 0.0018 | 0.751 |
| Activated CD69^+^/CD3^+^/CD4^-^   - PreOP | 0.052 ± 0.0046 | 0.040 ± 0.0075 | 0.198 |
| - POD1 | 0.046 ± 0.0050 | 0.046 ± 0.0090 | 1.0 ^b^ |
| - POW6 | 0.068 ± 0.0074 ^sig0/1^ | 0.056 ± 0.0074 | 0.388 |
|  | *[2^NPX^] (means ± SEM)* | |  |
| ICOS-L   - PreOP | 31.88 ± 1.28 | 37.02 ± 1.71 | 0.033 |
| - POW6 | 34.84 ± 1,35 | 35.51 ± 2.21 | 0.801 |
| FAS-L   - PreOP | 378.39 ± 36.30 | 625.34 ± 42.14 | 0.001 |
| - POW6 | 487.89 ± 36.00 | 601.95 ± 43.82 | 0.067 |
| CD207   - PreOP | 8.26 ± 0.52 | 12.32 ± 0.99 | 0.004 |
| - POW6 | 8.68 ± 0.57 | 10.91 ± 1.06 | 0.088 |
| CEACAM1 (CD66a)   - PreOP | 171.03 ± 4.96 | 149.60 ± 7.25 | 0.031 |
| - POW6 | 141.87 ± 6.72 ^sig0(d)^ | 148.90 ± 4.88 | 0.414 |
| CYR61   - PreOP | 92.49 ± 4.85 | 128.59 ± 14.28 | 0.036 |
| - POW6 | 94.04 ± 8.75 | 113.72 ± 15.41 | 0.289 |
| TRAIL   - PreOP | 178.72 ± 6.94 | 251.41 ± 17.40 | 0.002 |
| - POW6 | 199.52 ± 6.65 ^sig0(d)^ | 235.64 ± 13.42 | 0.097 ^b^ |
| MADH5   - PreOP | 13.52 ± 0.23 | 15.00 ± 0.38 | 0.006 |
| - POW6 | 14.42 ± 0.44 | 15.28 ± 0.67 | 0.306 |
| MIC-A/B   - PreOP | 46.37 ± 4.72 | 29.86 ± 3.96 | 0.007 ^b^ |
| - POW6 | 39.79 ± 4.69 | 29.23 ± 4.41 | 0.127 |
| IL6 (PEA)   - PreOP | 41.23 ± 32.66 | 8.58 ± 1.32 | 0.710 ^b^ |
| - POW6 | 19.08 ± 4.21 | 13.46 ± 4.25 | 0.365 |
|  | *[] (means ± SEM)* | |  |
| Stimulation ratio (CD69^+^/CD3^+^)   - PreOP | 6.617 ± 0.6149 | 9.913 ± 1.9290 | 0.039 |
| - POD1 | 5.071 ± 0.5456 ^sig0^ | 6.031 ± 0.9338 | 0.566^b^ |
| - POW6 | 6.143 ± 0.8316 | 7.026 ± 0.7117 | 0.539 |
|  | *[pg/ml] (means ± SEM)* | |  |
| IL2  (after in vitro stimulation)   - PreOP | 245.72 ± 42.15 | 420.63 ± 167.32 | 0.182 ^b^ |
| - POD1 | 181.52 ± 28.32 | 298.12 ± 75.67 | 0.199 ^b^ |
| - POW6 | 311.97 ± 60.65 ^sig1^ | 357.53 ± 93.10 | 0.497 ^b^ |
| IFNγ  (after in vitro stimulation)   - PreOP | 556.34 ± 126.25 | 492.76 ± 177.77 | 0.764 ^b^ |
| - POD1 | 432.45 ± 162.44 | 571.22 ± 165.91 | 0.317 ^b^ |
| - POW6 | 911.21 ± 288.03 | 682.68 ± 265.33 | 0.91 ^b^ |
| TNFα  (after in vitro stimulation)   - PreOP | 910.97 ± 137.72 | 1128.08 ± 192.29 | 0.182 ^b^ |
| - POD1 | 255.66 ± 33.84 ^sig0(c)^ | 469.49 ± 106.59 ^sig0(c)^ | 0.076 ^b^ |
| - POW6 | 1198.25 ± 137.26 ^sig1(c)^ | 1043.90±180.68 ^sig1(c)^ | 0.546 |
| IL10  (after in vitro stimulation)   - PreOP | 255.38 ± 43.60 | 162.82 ± 29.45 | 0.391 ^b^ |
| - POD1 | 182.12 ± 32.35 | 239.15 ± 55.71 | 0.417 ^b^ |
| - POW6 | 184.98 ± 33.66 | 179.52 ± 44.29 | 0.955 ^b^ |
| IL6  (serum concentration, ELISA)   - PreOP | 15.38 ± 6.17 | 44.0636 ± 24.18 | 0.417 ^b^ |
| - POD1 | 130.71 ± 28.44 ^sig0(c)^ | 83.6317 ± 28.51 ^sig0(c)^ | 0.417 ^b^ |
| - POW6 | 16.97 ± 8.22 ^sig1(c)^ | 53.2726 ± 33.44 | 0.141 ^b^ |

^a^ unpaired Student’s T-test, ^b^ Mann-Whitney-U-test, ^sig^ p<0.05 in repeated measures ANOVA and Bonferroni-corrected post-hoc test (^0^ compared with PreOP, ^1^ compared with POD1), ^sig(c)^ Friedmann-test (^0^ compared with PreOP, ^1^ compared with POD1), ^sig(d)^ paired samples T-test.

**Table S2.** Effect of whole blood stimulation with SEB on the expression of CD69 on CD3^+^, CD3^+^/CD4^+^ and CD3^+^/CD4^-^ lymphocytes.

|  | **Adenocarcinoma** | | | **Benign** | | |
| --- | --- | --- | --- | --- | --- | --- |
|  | **unstimulated**  **CD69** | **stimulated**  **CD69** | **p-value^d^** | **unstimulated**  **CD69** | **stimulated**  **CD69** | **p-value^d^** |
|  | *[relative proportion of 1] (means ± SEM)* | | | | | |
| CD3^+^ | 0.024 ± 0.011 | 0.145 ± 0.077 | <0.001 | 0.017 ± 0.005 | 0.152 ± 0.043 | <0.001 |
| CD3^+^  CD4^+^ | 0.012 ± 0.067 | 0.117 ± 0.067 | <0.001 | 0.010 ± 0.007 | 0.140 ± 0.042 | <0.001 |
| CD3^+^  CD4^-^ | 0.052 ± 0.023 | 0.216 ± 0.10 | <0.001 | 0.040 ± 0.020 | 0.184 ± 0.051 | 0.001 |

^d^ paired samples T-test.
